# Supplementary material for: Detection of SARS-CoV-2 intra-host recombination during superinfection with Alpha and Epsilon variants in New York City
Source: Nat Commun. 2022 Jun 25;13:3645. doi: 10.1038/s41467-022-31247-x (PMC9233664; doi:10.1038/s41467-022-31247-x)
Supplement: Supplementary file 1 — Supplementary Information [file 41467_2022_31247_MOESM1_ESM.pdf]

SUPPLEMENTARY INFORMATION:

Detection of SARS-CoV-2 intra-host recombination during superinfection with Alpha and Epsilon variants in New York City

J. Wertheim et al.

Supplementary Table 1. Recombinant haplotype frequencies across separate extractions, reverse transcription, and PCR amplification in high-throughput sequencing for 947 nt S gene fragment, nucleotide positions 23604 and 23709.

**Haplotypes**			**Count (frequencies)**			
**Type**	**23604**	**23709**	**NYCPHL-002461-A**	**NYCPHL-002461-B**	**NYCPHL-002461-C**	**NYCPHL-002461-D**
**MAJOR**	**A**	**T**	3633 (78.57%)	4095 (76.43%)	3778 (76.40%)	4745 (78.24%)
**MINOR**	**C**	**C**	634 (13.71%)	776 (14.48%)	762 (15.41%)	851 (14.03%)
**MIX**	**A**	**C**	162 (3.50%)	234 (4.37%)	191 (3.86%)	237 (3.91%)
**MIX**	**C**	**T**	195 (4.22%)	253 (4.72%)	214 (4.33%)	232 (3.83%)
**Depth**			4624 (100%)	5358 (100%)	4945 (100%)	6065 (100%)

Supplementary Table 2. Recombinant haplotype frequencies across separate extractions, reverse transcription, and PCR amplification in high-throughput sequencing for the 476 nt ORF8 fragment, nucleotide positions 27972, 28048, 28095, 28111.

Haplotypes					Count (frequencies)			
Type	27972	28048	28095	28111	NYCPHL-002461-A	NYCPHL-002461-B	NYCPHL-002461-C	NYCPHL-002461-D
MAJOR	T	T	T	G	1382 (70.22%)	1164 (69.83%)	1110 (68.90%)	1873 (70.26%)
MINOR	C	G	A	A	381 (19.36%)	305 (18.30%)	347 (21.54%)	505 (18.94%)
MIX	T	G	A	A	57 (2.90%)	40 (2.40%)	31 (1.92%)	77 (2.89%)
MIX	C	T	T	G	50 (2.54%)	60 (3.60%)	55 (3.41%)	66 (2.48%)
MIX	T	T	A	A	40 (2.03%)	43 (2.58%)	28 (1.74%)	48 (1.80%)
MIX	C	G	T	G	34 (1.73%)	28 (1.68%)	18 (1.12%)	45 (1.69%)
MIX	T	T	T	A	8 (0.41%)	7 (0.42%)	6 (0.37%)	12 (0.45%)
MIX	C	G	A	G	4 (0.20%)	6 (0.36%)	4 (0.25%)	12 (0.45%)
MIX	T	G	T	G	6 (0.30%)	8 (0.48%)	7 (0.43%)	15 (0.56%)
MIX	C	T	A	A	1 (0.05%)	3 (0.18%)	3 (0.19%)	5 (0.19%)
MIX	C	G	T	A	1 (0.05%)	1 (0.06%)	NULL	NULL
MIX	T	T	A	G	1 (0.05%)	1 (0.06%)	2 (0.12%)	7 (0.26%)
MIX	C	T	A	G	1 (0.05%)	1 (0.06%)	NULL	NULL
MIX	T	G	T	A	1 (0.05%)	NULL	NULL	NULL
MIX	C	T	T	A	1 (0.05%)	NULL	NULL	1 (0.04%)
Depth					1968 (100%)	1667 (100%)	1611 (100%)	2666 (100%)

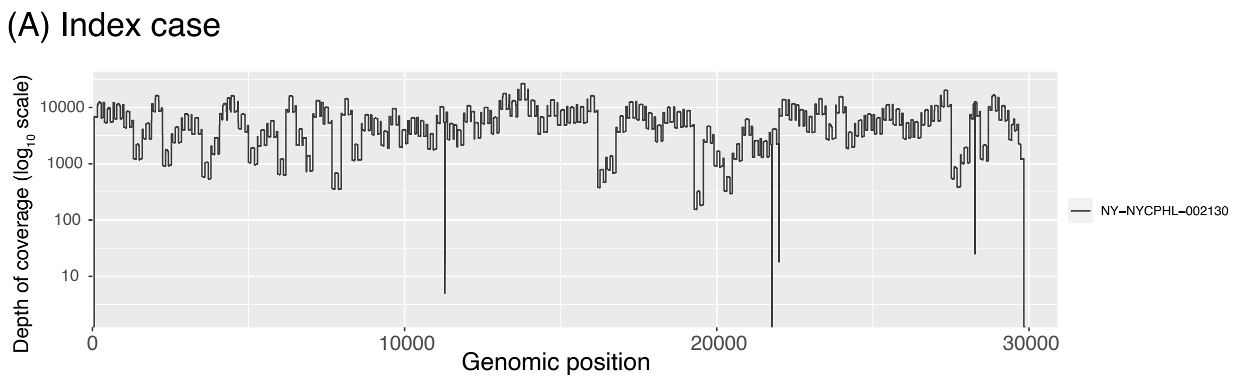

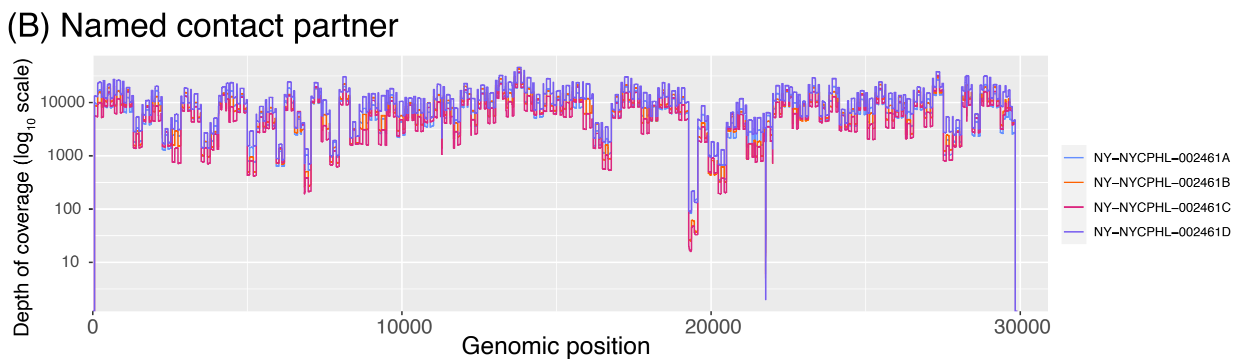

Supplementary Figure 1. Genomic sequencing depth in the (A) index case NY-NYCPHL-002130 and (B) named contact partner NY-NYCPHL-002461.
