# Supplementary material for: Detection of SARS-CoV-2 intra-host recombination during superinfection with Alpha and Epsilon variants in New York City
Source: Nat Commun. 2022 Jun 25;13:3645. doi: 10.1038/s41467-022-31247-x (PMC9233664; doi:10.1038/s41467-022-31247-x)
Supplement: Supplementary file 2 — Description of Additional Supplementary Files [file 41467_2022_31247_MOESM2_ESM.docx]

File Name: Supplementary Data 1

Description: List acknowledging the authors who submitted the SARS-CoV-2 B.1.1.7 (Alpha) and B.1.429 (Epsilon) genomes (n=5934) analyzed in this study.
