# Supplementary material for: Detection of SARS-CoV-2 intra-host recombination during superinfection with Alpha and Epsilon variants in New York City
Source: Nat Commun. 2022 Jun 25;13:3645. doi: 10.1038/s41467-022-31247-x (PMC9233664; doi:10.1038/s41467-022-31247-x)
Supplement: Supplementary file 4 — Reporting Summary [file 41467_2022_31247_MOESM4_ESM.pdf]

nature portfolio

Corresponding author(s): Joel Wertheim
Jade Wang

Last updated by author(s): May 19, 2022

Reporting Summary

Nature Portfolio wishes to improve the reproducibility of the work that we publish. This form provides structure for consistency and transparency in reporting. For further information on Nature Portfolio policies, see our Editorial Policies and the Editorial Policy Checklist.

Statistics

For all statistical analyses, confirm that the following items are present in the figure legend, table legend, main text, or Methods section.

n/a	Confirmed	
☐	☒	The exact sample size (*n*) for each experimental group/condition, given as a discrete number and unit of measurement
☐	☒	A statement on whether measurements were taken from distinct samples or whether the same sample was measured repeatedly
☐	☒	The statistical test(s) used AND whether they are one- or two-sided<br>*Only common tests should be described solely by name; describe more complex techniques in the Methods section.*
☒	☐	A description of all covariates tested
☒	☐	A description of any assumptions or corrections, such as tests of normality and adjustment for multiple comparisons
☒	☐	A full description of the statistical parameters including central tendency (e.g. means) or other basic estimates (e.g. regression coefficient) AND variation (e.g. standard deviation) or associated estimates of uncertainty (e.g. confidence intervals)
☐	☒	For null hypothesis testing, the test statistic (e.g. *F*, *t*, *r*) with confidence intervals, effect sizes, degrees of freedom and *P* value noted<br>*Give P values as exact values whenever suitable.*
☒	☐	For Bayesian analysis, information on the choice of priors and Markov chain Monte Carlo settings
☒	☐	For hierarchical and complex designs, identification of the appropriate level for tests and full reporting of outcomes
☒	☐	Estimates of effect sizes (e.g. Cohen's *d*, Pearson's *r*), indicating how they were calculated

Our web collection on statistics for biologists contains articles on many of the points above.

Software and code

Policy information about availability of computer code

Data collection	No custom software was used to collect these data
Data analysis	These data analyzed using publicly available software: RDP5, TreeTime v0.8.0, Mafft v7.464, IQTree2 v2.1.3, Galaxy SARS-CoV-2 Allelic Variation Pipeline v0.5

For manuscripts utilizing custom algorithms or software that are central to the research but not yet described in published literature, software must be made available to editors and reviewers. We strongly encourage code deposition in a community repository (e.g. GitHub). See the Nature Portfolio guidelines for submitting code & software for further information.

Data

Policy information about availability of data

All manuscripts must include a data availability statement. This statement should provide the following information, where applicable:

- Accession codes, unique identifiers, or web links for publicly available datasets
- A description of any restrictions on data availability
- For clinical datasets or third party data, please ensure that the statement adheres to our policy

The data analyzed as part of this project were obtained from the GISAID database and through a Data Use Agreement between NYC DOHMH and the University of California San Diego. We gratefully acknowledge the authors from the originating laboratories and the submitting laboratories, who generated and shared via GISAID the viral genomic sequence data on which this research is based. A complete list acknowledging the authors who submitted the data analyzed in this study can be found in Data S1.

Trimmed, host-depleted viral sequencing data and cloned sequence fragments have been submitted to NCBI (accession numbers pending).

Field-specific reporting

Please select the one below that is the best fit for your research. If you are not sure, read the appropriate sections before making your selection.

☐ Life sciences ☐ Behavioural & social sciences ☒ Ecological, evolutionary & environmental sciences

For a reference copy of the document with all sections, see nature.com/documents/nr-reporting-summary-flat.pdf

Ecological, evolutionary & environmental sciences study design

All studies must disclose on these points even when the disclosure is negative.

Study description	This study analyzed samples collected as part of routine SARS-CoV-2 public health surveillance. Full and partial genome sequencing was performed on these samples and genetic and phylogenetic anan
Research sample	Two individuals were identified within the New York City Department of Health and Mental Hygiene SARS-CoV-2 surveillance database sampled in January 2021. These data were analyzed in the context of SARS-CoV-2 genomes available on GISIAD.
Sampling strategy	No sampling strategy was employed due to the nature of infectious disease public health surveillance
Data collection	Primary samples were collected in January 2021 as part of routine public health surveillance in New York City through the Electronic Clinical Laboratory Reporting System (ECLRS)
Timing and spatial scale	Primary samples were collected in January 2021 as part of routine public health surveillance in New York City; data from November 2020 through March 2021 were included
Data exclusions	Data exclusion is not appropriate of infectious disease surveillance
Reproducibility	Nucleotide extraction and genome sequencing was performed in quadruplicate; all extractions were successful
Randomization	Randomization is not appropriate for infectious disease surveillance
Blinding	Blinding is not appropriate for infectious disease surveillance

Did the study involve field work? ☐ Yes ☒ No

Reporting for specific materials, systems and methods

We require information from authors about some types of materials, experimental systems and methods used in many studies. Here, indicate whether each material, system or method listed is relevant to your study. If you are not sure if a list item applies to your research, read the appropriate section before selecting a response.

Materials & experimental systems

n/a	Involved in the study	
☒	☐	Antibodies
☒	☐	Eukaryotic cell lines
☒	☐	Palaeontology and archaeology
☒	☐	Animals and other organisms
☐	☒	Human research participants
☒	☐	Clinical data
☒	☐	Dual use research of concern

Methods

n/a	Involved in the study	
☒	☐	ChIP-seq
☒	☐	Flow cytometry
☒	☐	MRI-based neuroimaging

Human research participants

Policy information about studies involving human research participants

Population characteristics	The population from which these samples were identified represent all people who tested-positive for SARS-CoV-2 using a PCR-assay at a lab operated by the New York City Department of Health and Mental Hygiene. This analysis is considered Human-Subjects Except, as all data were deidentified prior to sharing with outside researchers
Recruitment	No recruitment took place as part of this study
Ethics oversight	UC San Diego IRB approved the analysis of deidentified SARS-CoV-2 public health surveillance data

Note that full information on the approval of the study protocol must also be provided in the manuscript.
